# Micro-Raman Spectroscopy Reveals Compositional Alterations in Molar Incisor Hypomineralization and Enamel Adjacent to Demarcated Opacities

**DOI:** 10.1007/s00223-026-01517-7

**Published:** 2026-04-27

**Authors:** Flaureta Rexhaj, Furqan A. Shah, Ted Lundgren

**Affiliations:** 1 Department of Pediatric Dentistry, Institute of Odontology, Sahlgrenska Academy, University of Gothenburg, Göteborg, Sweden; 2 Teaching Clinic for Pediatric Dentistry, Public Dental Service, University Dental Care, Region Västra Götaland, Gothenburg, Sweden; 3 Department of Biomaterials, Institute of Clinical Sciences, Sahlgrenska Academy, University of Gothenburg, Göteborg, Sweden

**Keywords:** Dental enamel, Raman spectroscopy, Children, Hypomineralized teeth, Molar incisor hypomineralization

## Abstract

Molar incisor hypomineralization (MIH) is a developmental condition that impairs enamel mineralization in the first permanent molars and often the permanent incisors. Clinically, MIH presents as demarcated opacities, ranging from white–yellow discolorations in mild cases to yellow–brown discolorations with enamel breakdown in severe cases. The longevity of direct adhesive restorations depends on reliable bonding to sound enamel, and current clinical practice involves removing visibly affected enamel and bonding adhesive restorations to apparently sound tissue. Accurately identifying healthy enamel is therefore crucial. Despite increasing recognition of MIH, there remains a gap in understanding its pathogenesis and the optimal treatment strategies needed to ensure long-term restorative success. The enamel adjacent to demarcated opacities has not been thoroughly investigated, raising a critical question: does visually translucent, seemingly intact enamel next to MIH opacities truly represent unaffected tissue? This work uses micro-Raman spectroscopy to determine whether chemical and structural alterations extend beyond the margins of demarcated opacities into adjacent translucent enamel. The findings indicate that MIH involves not only the visibly opaque enamel but also the adjacent translucent enamel, which shows increased fluorescence, lower mineral crystallinity, and elevated acid phosphate and carbonate content. These substitutions are consistent with a more soluble and porous apatite phase that is less chemically durable in the physiological environment. The adjacent translucent region consistently occupies an intermediate position between healthy controls and opacities, supporting its classification as affected tissue, and highlighting implications for adhesive strategies, margin placement, and preventive management of MIH-affected teeth.

## Introduction

Tooth enamel is the hardest and most highly mineralized tissue in the human body. It consists of approximately 95 wt% inorganic material, mainly hydroxyapatite or hydroxy(l)apatite (HAp), 1–2 wt% organic material, and 2–4 wt% water [1, 2]. Bundles of highly crystalline HAp nanowires are organized into tightly packed enamel rods 4–5 μm in diameter [3], which extend from the dentin-enamel junction (DEJ) to the external surface of the tooth. The interrod substance between enamel rods contains enamel-specific proteins, of which amelogenin is the most abundant [4]. The mechanical strength and wear resistance of enamel are governed by factors such as the size, order, and alignment of HAp crystals and the balance between the organic and inorganic components [1].

Molar incisor hypomineralization (MIH) is a developmental enamel defect of first permanent molars and often incisors, characterized by demarcated opacities [5]. MIH enamel is porous, and the degree of porosity relates to opacity colour, with white-to-yellow opacities being less porous and yellow-to-brown being more porous and severely affected [6]. MIH affects one in six children worldwide [7] and causes hypersensitivity that may lead to dental fear, impaired eating and toothbrushing, complex clinical management, reduced quality of life, and increased healthcare costs and resource demands for individuals and society [8].

The etiology of MIH is unclear, but evidence supports a multifactorial origin involving genetic, environmental, and systemic disturbances during the prenatal or early postnatal period, when enamel of molars and incisors forms [9]. During amelogenesis, the organic matrix is degraded and replaced by mineral, yielding enamel with less than 1% residual organic material [2, 4]. Disruption of this process produces hypomineralized enamel with altered structure and composition, and in MIH, the organic content is significantly higher than in healthy enamel [10, 11].

MIH pathogenesis and evidence-based strategies for long-term restorative success remain poorly understood. The longevity of direct adhesive restorations depends on a durable bond to sound enamel, underscoring the need to identify and preserve healthy tissue [12]. Clinically, MIH teeth are treated by removing visibly affected enamel and bonding restorations to sound enamel [13]. However, enamel beyond visible defects is unclear, raising questions about the structural and chemical integrity of adjacent visually translucent enamel [14–16]. Previous studies have mainly characterized the demarcated opacities [17–20]. This uncertainty prompts the question of whether visually translucent enamel next to MIH opacities truly represents unaffected, sound enamel.

The composition and structure of dental tissues can be analyzed non-destructively using Raman spectroscopy [21]. It has been used for bone, enamel defects (caries, fluorosis), mineralized dental biofilms, and developmental enamel defects including MIH [19, 20, 22–28]. Raman studies show increased organic content and higher carbonate substitution in MIH enamel [20]. Raman spectroscopy can detect compositional changes in early demineralization before visible damage [29], highlighting the potential of this technique to characterize MIH and surrounding enamel, investigate enamel biology and pathology, and support the development of treatment strategies and more durable dental materials.

Considering that MIH is a developmental defect, this work uses micro-Raman spectroscopy to determine whether chemical and structural alterations in enamel extend beyond the visible margins of demarcated opacities in MIH-affected teeth. Specifically, we test the hypothesis that translucent enamel adjacent to MIH opacities differs from sound enamel in composition and structure, and that such alterations may help explain the increased failure rates of adhesive restorations in MIH-affected teeth [30].

## Materials and Methods

### Study Population and Tooth Selection

Children aged 6–13 years diagnosed with MIH according to the European Academy of Paediatric Dentistry criteria were recruited. Teeth scheduled for extraction were considered for donation. Eleven permanent first molars from ten children with MIH were included (one child contributed two teeth), each showing white-to-yellow demarcated opacities on occlusal enamel. Healthy teeth were obtained from children without underlying dental or systemic conditions, and comprised two clinically sound premolars and one molar extracted for orthodontic indications. All were assessed as free from caries, restorations, and mineralization defects.

### Ethical Considerations

The Central Ethical Review Board, Gothenburg, Sweden, approved the study (Dnr 924−16). The study was conducted in accordance with the World Medical Association Declaration of Helsinki. Children and their parents were informed about the aim and study design. Written and informed consent was obtained from parents, and assent was obtained from the children before donation of extracted teeth.

### Sample Handling and Surface Preparation

Immediately after removal, teeth were immersed in 70% ethanol and stored at room temperature. Demarcated opacities and adjacent translucent enamel were identified by a pediatric dentist using a stereo microscope (Leica M80) at ×20 magnification. To remove surface debris (e.g., mineralized biofilm), the surface was etched twice with 35% phosphoric acid for 30 s, rinsed with tap water, and then cleaned with a soft polishing brush.

### Backscattered Electron Scanning Electron Microscopy (BSE-SEM)

To verify that the cleaning protocol effectively eliminated mineralized biofilm or other debris on the tooth surface, backscattered electron scanning electron microscopy (BSE-SEM) was performed in a Quanta 200 environmental SEM (FEI Company, The Netherlands) operated at 1 Torr water vapor pressure and 20 kV accelerating voltage.

### Micro-Raman Spectroscopy

Micro-Raman spectroscopy was performed using a confocal Raman microscope (Renishaw inVia Qontor, Renishaw plc. Wotton-under-Edge, UK) equipped with a 785 nm laser and LiveTrack™ focus-tracking technology. The laser was focused onto the sample surface using a 50×/0.5 NA objective. The Raman scattered light was collected using a Peltier-cooled CCD deep depletion NIR enhanced detector behind a 1200 g mm^− 1^ grating (347–1507 cm^–1^ wavenumber range) at a pixel size of 50 μm × 50 μm and integration time of 2 s per pixel. The laser power at the sample was ~85 mW.

On control and MIH-affected teeth, Raman maps contained 240–300 spectra as 3–5 lines (50–80 spectra per line at a step size of 50 μm). On control teeth, the ROIs were positioned on *healthy enamel* (HE) free from occlusal wear. On MIH-affected teeth, the ROIs were positioned across the interface between *translucent enamel* (TE) and *opaque enamel* (OE). Baseline correction and cosmic ray removal were performed in Renishaw WiRE 5.4 software. For MIH-affected teeth, TE and OE zones were delineated using the freehand mask tool. Average spectra were obtained for each TE and OE zone (*n* = 11). One average spectrum was obtained for each control tooth (*n* = 3).

Signal-to-noise ratios (SNR) were computed as the ratio between the ν_2_ PO_4_^3−^ (410–470 cm^− 1^) or ν_1_ PO_4_^3−^ (940–980 cm^− 1^) integral area and the integral area of the fluorescence background (subtracted baseline) over the same wavenumber ranges. Here, noise refers to the sample-dependent fluorescence background, rather than stochastic detector noise. Mineral crystallinity was taken as the inverse full-width at half-maximum (FWHM) of the ν_1_ PO_4_^3−^ band at 960 cm^− 1^. FWHM ν_1_ PO_4_^3−^ was obtained by non-linear least-squares (NLLS) curve fitting. The HPO_4_^2−^ or acid phosphate content (HPO_4_^2−^/ ν_1_ PO_4_^3−^ intensity ratio) was estimated from the 880/960 cm^− 1^ ratio [31]. The CO_3_^2−^ content (ν_1_ CO_3_^2−^/ν_1_ PO_4_^3−^ intensity ratio) was determined from the 1070/960 cm^− 1^ ratio.

### Statistical Analysis

The Wilcoxon signed-rank test was used for pairwise comparisons between OE and TE, and the Mann-Whitney U test was used for comparisons between TE or OE and HE, and *p* values < 0.05 were considered statistically significant. Mean values ± standard deviations are shown.

## Results

### Heterogeneous Microstructure in Translucent Enamel

**Fig. 1:**
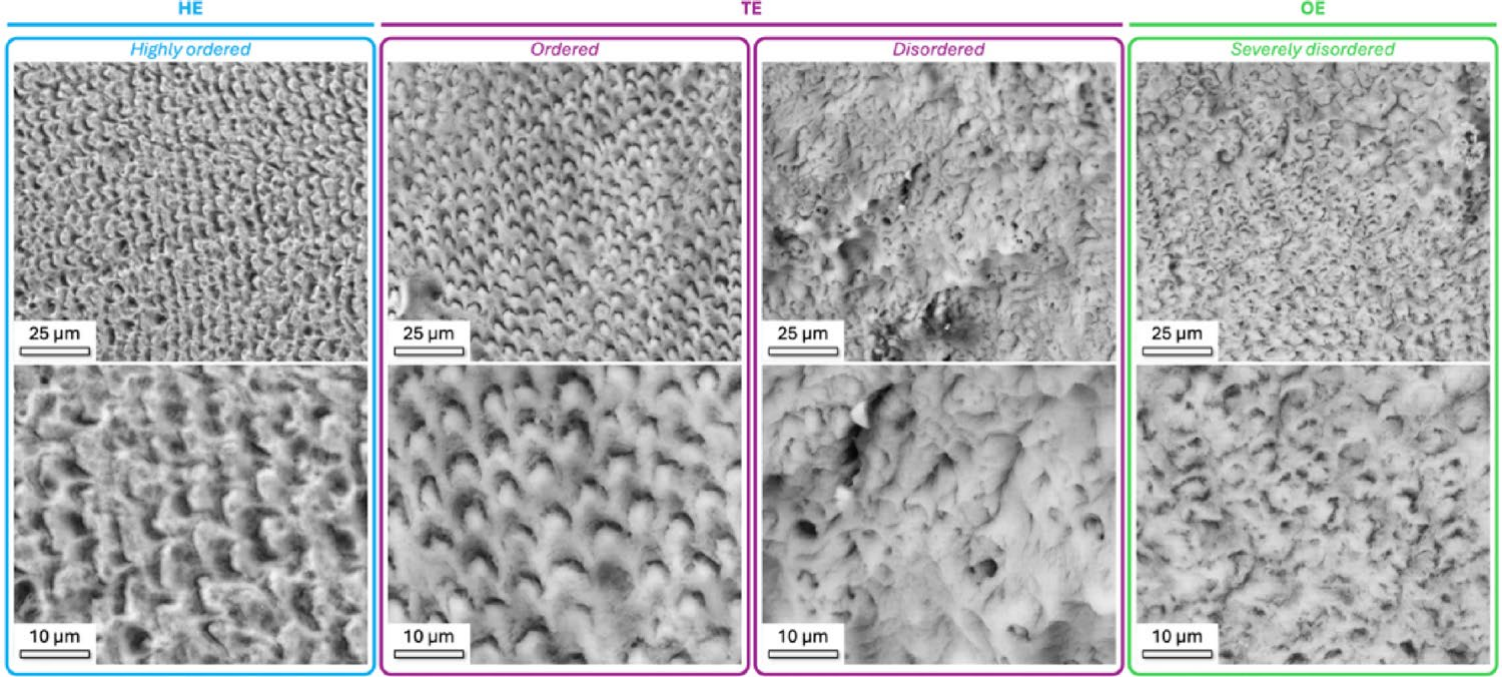
Occlusal surface of healthy and MIH-affected teeth observed with BSE-SEM. HE shows highly ordered enamel rod and interrod spaces. TE is heterogeneous and varies between regions with ordered enamel rods and regions with moderately disordered rods and interrod spaces. OE on MIH teeth is characterized by severe disorder with indistinct rod and interrod spaces

BSE-SEM reveals distinct differences in microstructural organization between HE, TE and OE, highlighting a gradual disruption of enamel microarchitecture associated with MIH (Fig. 1). HE is characterized by highly ordered, clearly discernible enamel rods and interrod spaces. TE varies between two extreme appearances, varying between regions that are well-ordered and regions that contain moderately disordered rods and interrod spaces. OE shows severe structural disorder, with indistinct rods and interrod spaces. A mineralized biofilm was not observed on the enamel surface confirming the efficacy of the cleaning protocol.

### Surface-tracked Raman Spectroscopy Reveals Spatial Chemical Gradients

**Fig. 2:**
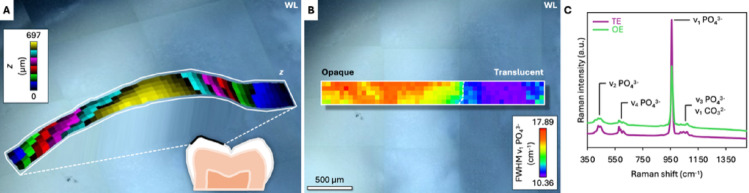
Real-time surface tracked micro-Raman spectroscopy of enamel at the occlusal surface. The example shows one MIH tooth. **A** Height deviation (z) map of the native tooth surface. The highest point in the map corresponds to the cusp tip, ~700 μm above the lowest point in the map. **B** Corresponding Raman map of FWHM ν_1_ PO_4_^3−^ (940–980 cm^−1^ range) across the interface between opaque enamel (OE) and translucent enamel (TE) on the occlusal surface, indicated by the white broken line. FWHM ν_1_ PO_4_^3−^ is inversely related to mineral crystallinity (i.e., higher values indicate lower crystallinity). WL = white light image. **C** Raman spectra of TE (average of 92 pixels) and OE (average of 158 pixels)

Surface-tracked Raman spectroscopy enabled seamless chemical mapping over the native, non-planar enamel surface (Fig. 2A), and allowed chemical information to be correlated with the optical appearance of OE and TE in white light images (Fig. 2B, C).

### Elevated Fluorescence and Reduced Raman Signal-to-Noise Ratio in MIH

**Fig. 3:**
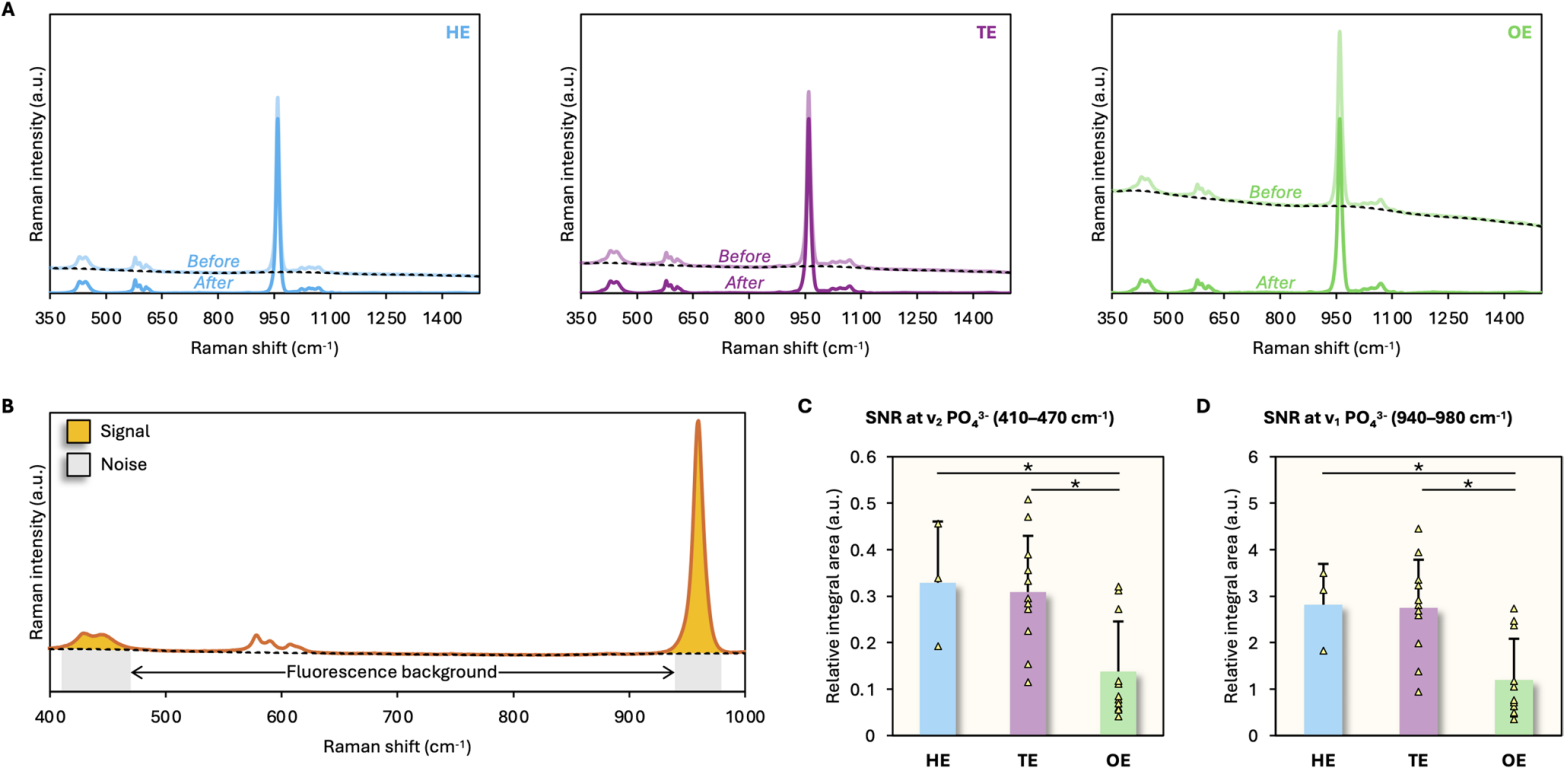
Raman signal quality differs between HE, TE, and OE. **A** Averaged Raman spectra of HE (*n* = 3), TE (*n* = 11), and OE (*n* = 11) before and after background subtraction. Broken line indicates the background fluorescence profile. **B** SNR is taken as the ratio between the Raman integral area and the subtracted fluorescence signal over the corresponding wavenumber range. **C** SNR for HE, TE, and OE at 410–470 cm^−1^. **D** SNR for HE, TE and OE at 940–980 cm^−1^. * = Statistically significant

HE, TE, and OE showed a broad fluorescence background. After background subtraction, the underlying Raman bands were still clearly visible. The relative contribution of fluorescence was slightly higher in TE and markedly higher in OE (Fig. 3A). Consequently, the SNR at ν_2_ PO_4_^3−^ (410–470 cm^−1^) and ν_1_ PO_4_^3−^ (940–980 cm^−1^) is significantly lower for OE, compared to TE (*p* = 0.0033) and HE (*p* = 0.0384) (Fig. 3B–D).

### MIH Enamel Shows Reduced Mineral Crystallinity

**Fig. 4:**
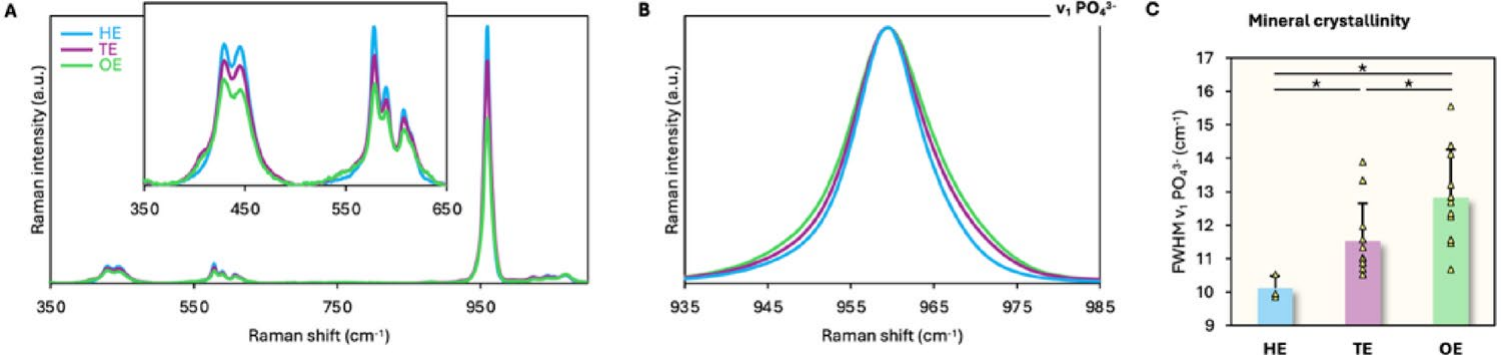
ν_1_, ν_2_, and ν_4_ PO_4_^3−^ bands in HE, TE, and OE. **A** ν_1_ PO_4_^3−^ band intensity progressively decreases between HE, TE, and OE. Inset: Detail of the 350–650 cm^−1^ region, showing the ν_2_ PO_4_^3−^ (390–490 cm^−1^) and ν_4_ PO_4_^3−^ (500–650 cm^−1^) bands. **B** ν_1_ PO_4_^3−^ band broadens progressively between HE, TE, and OE. Normalized spectra. **C** FWHM ν_1_ PO_4_^3−^. * = Statistically significant

MIH enamel, i.e., TE and OE, and HE show similar spectral features. After background subtraction, all phosphate bands (ν_1_ PO_4_^3−^, ν_2_ PO_4_^3−^, and ν_4_ PO_4_^3−^) decreased progressively from HE to TE and OE. In particular, the ν_1_ PO_4_^3−^ band was 13% lower in TE and ~36% lower in OE, compared to HE (Fig. 4A). The ν_2_ PO_4_^3−^ and ν_4_ PO_4_^3−^ band profiles of TE and OE are similar to HE, and characteristic of enamel. The ν_2_ PO_4_^3−^ band comprises sub-components at 428 cm^−1^ and 450 cm^−1^ of approximately similar intensity. The ν_4_ PO_4_^3−^ band comprises sub-components at 578 cm^−1^, 590 cm^−1^, and 607 cm^−1^, of which the 578 cm^−1^ and 590 cm^−1^ sub-components are clearly resolved. The ν_1_ PO_4_^3−^ band shows progressive broadening from HE to TE and OE Fig. 4B). Compared to HE, both OE (*p* = 0.0054) and TE (*p* = 0.0219) show higher FWHM ν_1_ PO_4_^3−^ (Fig. 4C**)**. OE shows higher FWHM ν_1_ PO_4_^3−^ than TE (*p* = 0.0098).

### MIH Enamel is Enriched in Acid Phosphate and Carbonate

**Fig. 5:**
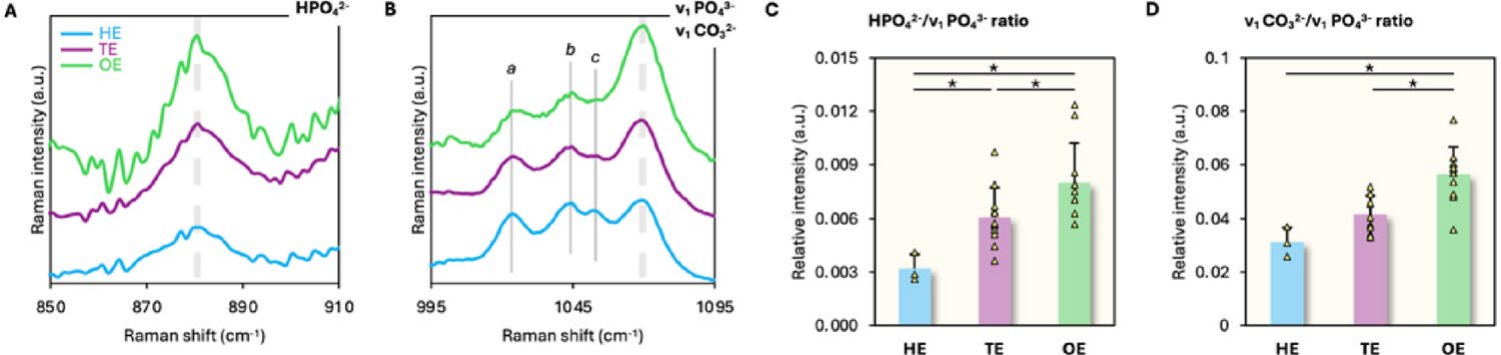
HPO_4_^2−^ and ν_3_ PO_4_^3−^–ν_1_ CO_**3**_^2−^ regions of HE, TE, and OE. **A** The HPO_4_^2−^ or acid phosphate region (broken line) of HE, TE, and OE. Normalized spectra; vertically offset for clarity. **B** The ν_3_ PO_4_^3−^–ν_1_ CO_3_^2−^ region (broken line) of HE, TE, and OE. Features labelled a, b, and c are assigned as ν_3_ PO_4_^3−^. Normalized spectra; vertically offset for clarity. **C** HPO_4_^2−^/ν_1_ PO_4_^3−^ ratio. **D** ν_1_ CO_3_^2−^/ν_1_ PO_4_^3−^ ratio. * = Statistically significant

The HPO_4_^2−^/ ν_1_ PO_4_^3−^ and CO_3_^2−^/PO_4_^3−^ ratios increase from HE to TE and are highest in OE. (Fig. 5A, B). The HPO_4_^2−^ content of TE (*p* = 0.0109) and OE (*p* = 0.0054) is higher than HE, where OE consistently shows higher HPO_4_^2−^ content than TE (*p* = 0.0466) (Fig. 5C). The CO_3_^2−^ content of TE tended to be higher than HE (*p* > 0.05), whereas OE shows significantly higher CO_3_^2−^ content than HE (*p* = 0.010989) and TE (*p* = 0.00694) (Fig. 5D).

### Minor Organic Inclusions in MIH Enamel

**Fig. 6:**
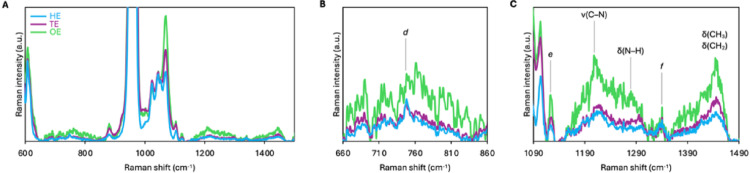
Organic inclusions in HE, TE, and OE. **A** The 600–1500 cm^−1^ region shows various organic signatures. Normalized spectra. **B** Detail of the 660–860 cm^−1^ region. Feature labelled d is assigned as a porphyrinic/heme-related peak. Normalized spectra. **C** Detail of the 1090–1490 cm^−1^ region. Features labelled e and f are assigned as porphyrinic/heme-related peaks. Normalized spectra

Several features were detected within the 650–1500 cm^−1^ range, attributed to organic moieties (≤ 1% of ν_1_ PO_4_^3−^ peak intensity) (Fig. 6A). A small band at ~750 cm^−1^, together with ~1122 cm^−1^ and ~1338 cm^−1^, forms a characteristic porphyrinic triplet consistent with heme or cytochrome-type vibrations (Fig. 6B). In addition, an amide III doublet at ~1210 cm^−1^ and **~**1285 cm^−1^, corresponding primarily to ν(C–N) and δ(N–H) vibrations of the peptide backbone, was observed. A ~1440 cm^−1^ band assigned to δ(CH_3_) and δ(CH_2_) deformation modes of methylene and methyl groups in proteins and lipids was also present (Fig. 6C).

## Discussion

**Fig. 7:**
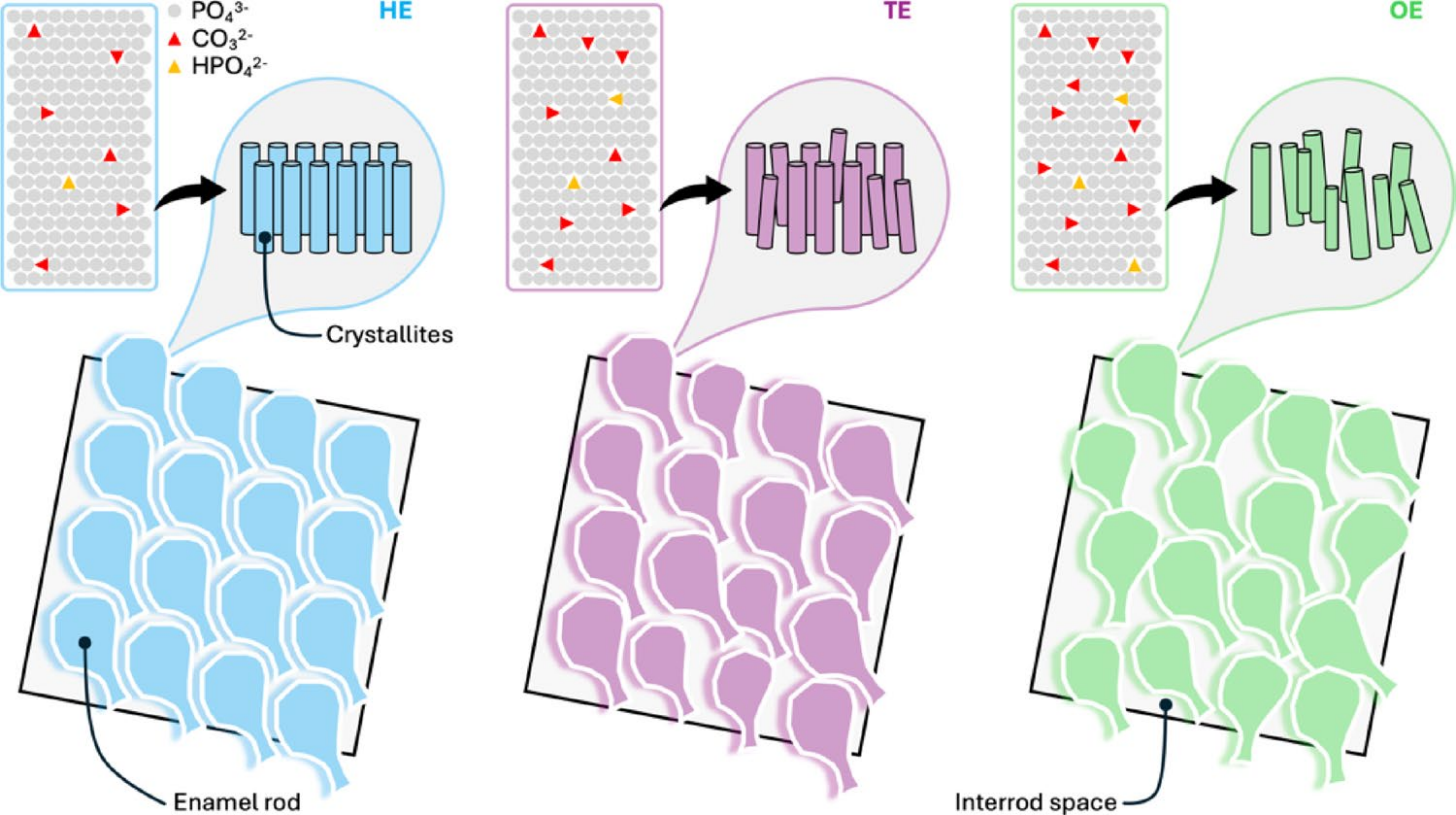
Illustration of enamel composition, crystallinity, and rod–interrod space relationships in HE, TE, and OE. Compared to HE, MIH enamel (i.e., TE and OE) shows higher apparent HPO_4_^2−^ and CO_3_^2−^ substitution at PO_4_^3−^ sites, resulting in increased lattice strain, decreased mineral crystallinity, and disordered enamel rod–interrod relationships

In this work, we used micro-Raman spectroscopy to test whether translucent enamel (TE) adjacent to opaque enamel (OE) deviates from healthy enamel (HE) in composition and structure. Visually sound TE surrounding demarcated opacities on MIH teeth shows measurable alterations (Fig. 7). Adjacent TE showed higher fluorescence, lower SNR, ν_1_ PO_4_^3−^ peak broadening, and increased HPO_4_^2−^ and CO_3_^2−^ content. Across Raman-derived metrics, TE values were intermediate between HE and OE, suggesting that MIH-related alterations extend beyond the visually demarcated lesion. Higher FWHM indicates decreased crystallinity (greater lattice disorder). Broadening of the ν_1_ PO_4_^3−^ band is commonly used as an indicator of lattice disorder and lower crystallinity in geologic, biogenic, and synthetic apatites. Relative to enamel on clinically sound teeth, ν_1_ PO_4_^3−^ broadened by ~27% in OE and ~14% in TE.

Overall, MIH enamel showed lower mineral crystallinity with higher HPO_4_^2−^ and CO_3_^2−^ content. These changes are consistent with poorly crystalline carbonated apatite, where a key step in mineral maturation may have been disturbed. HAp crystals of enamel initially form in the presence of protons (H^+^) and water, which leads to the partial protonation of phosphate groups and incorporation of HPO_4_^2−^ alongside PO_4_^3−^ either into the crystal lattice or at crystal surfaces. Elevated HPO_4_^2−^ content (i.e., HPO_4_^2−^/ν_1_ PO_4_^3−^ ratio) is consistent with apatite formed under a more acidic microenvironment, as may be expected if the maturation stage of amelogenesis is disrupted. At neutral-to-alkaline pH, deprotonation favors PO_4_^3−^ and produces more crystalline apatite, whereas at lower pH, more H^+^ ions are available, driving protonation and resulting in less crystalline apatite [32, 33].

The higher CO_3_^2−^/PO_4_^3−^ ratio of TE and OE is consistent with B-type carbonated apatite [34], presumably as post-amelogenetic deposition into tooth surface irregularities, when the environmental conditions (e.g., pH and availability of relevant ionic species) are conducive. Since CO_3_^2−^ substitution of PO_4_^3−^ introduces disorder into the apatite lattice [35], the presence of CO_3_^2−^-rich apatite as a secondary phase, localized to the crystal surface or grain boundaries [36], in addition to HPO_4_^2−^-enriched apatite, helps to explain the overall decrease in mineral crystallinity. Elevated HPO_4_^2−^ and CO_3_^2−^ substituting for PO_4_^3−^ not only reduce the chemical durability of the apatite phase but also disturb lattice charge balance. This interpretation is consistent with solubility studies on synthetic calcium phosphates, which show that carbonated, calcium-deficient and poorly crystalline apatites dissolve more readily than stoichiometric HAp. This may lead to calcium vacancies and/or charge compensation by additional anions [37], which further increases structural disorder and undermines mineral stability.

This interpretation aligns with prior reports of altered elemental distributions in MIH enamel (e.g., magnesium and chloride) relative to clinically normal enamel [16]. Together, these findings support a transient disturbance during amelogenesis that produces porous, chemically altered MIH enamel.

Our findings agree with reports of higher B-type carbonate and reduced crystallinity in MIH enamel [19, 20], and extend the changes to lesion-adjacent TE, showing that this visually sound zone shows measurable chemical and structural deviation from healthy controls. Observations on ribbon-like hypomineralization (RLH) [28] further highlight heterogeneity among enamel defects; unlike MIH, RLH has been described as carbonate-poor, indicating that not all hypomineralization phenotypes share the same mineral-chemical signature.

Compared with presumably unaffected enamel in the cervical area [17], the mechanical properties (e.g., elastic modulus, hardness, and flexural strength) are not only decreased at the opaque enamel on MIH teeth but also at the adjacent optically translucent enamel, albeit to a lesser extent [14]. Experimentally, it is difficult to universally benchmark how far these alterations extend from the OE-TE interface, given the irregular lesion geometry and surface topography. Nevertheless, the graded changes from HE to TE to OE in our Raman data align with mechanical observations [15] and support a peri-lesional altered zone with progressively compromised material properties between HE, TE, and OE.

In addition to mineral-related changes, MIH enamel also contained minor organic signatures. Within the 650–1500 cm^− 1^ range, we observed a weak porphyrinic triplet (~750, ~1122, and ~1338 cm^− 1^) consistent with heme- or cytochrome-type vibrations, together with an amide III doublet (~1210 and ~1285 cm^− 1^) and a δ(CH_3_), δ(CH_2_) deformation band (~1440 cm^− 1^). Although low in intensity (≤ 1% of the phosphate peak intensity), these bands are consistent with small amounts of retained or entrapped organic material within porous and structurally disordered MIH enamel [38, 39].

Besides the Raman bands typically examined for bioapatite, SNR primarily captures fluorescence-driven background interference and therefore serves as a practical index of Raman spectral quality across regions. At the demarcated opacities, lower SNR corresponds to increased fluorescence, which has been attributed to endogenous fluorophores such as porphyrins in other mineralized tissues [40–42], and may be consistent with organic moieties entrapped in the porous and/or disordered structure of MIH-affected enamel. While not a specific assay for organics, the concordance of fluorescence with ν_1_ PO_4_^3−^ FWHM suggests an increased background contribution in MIH regions, potentially including extrinsic sources (pellicle, microbial biofilm, or stains).

A key limitation is MIH heterogeneity, with variability between teeth, between lesions, and within lesions, which presents challenges for generalizing from small (or convenience-based) sample sets. Limited sample size (including that in the number of healthy teeth) can reduce statistical power, meaning that subtle compositional differences or spatial trends may remain undetected. Given the small number of healthy teeth, HE is only used as an external reference to contextualize whether TE values fall within the non-affected enamel range.

Furthermore, the present data are not directly correlated with mechanical or adhesive performance, nor is the organic content quantified by independent assays. Future work should, therefore, include larger cohorts with severity-based stratification, incorporate proteomic assays, and integrate mechanical and adhesive testing to relate chemistry to functional behavior. The spatial extent of MIH-related alterations also remains incompletely resolved, particularly the lateral distance from a demarcated opacity over which nominally translucent enamel exhibits measurable changes. Addressing this will be essential for defining clinically relevant margins such as for restorative procedures [14, 15] and improving treatment planning.

Our Raman measurements describe the chemistry of the near-surface enamel (< 10 μm in depth). Consequently, compositional features or gradients substantially below the enamel surface were not captured. However, the current setup allowed non-destructive examination (i.e., without sectioning), preserving the native optical characteristics of the enamel. Therefore, the Raman chemical maps could be directly correlated with the optical appearance of the same areas. Features such as increased opacity, loss of translucency, and locally altered color, which are hallmarks of MIH on clinical inspection, were linked to underlying chemical differences revealed by Raman spectroscopy.

Real-time focus tracking enabled mapping on non-planar enamel surfaces, without sectioning and thus preserving the native topography and optical appearance of MIH-affected enamel. The within-tooth comparison between OE and TE reduced inter-subject variability and improved sensitivity to subtle changes. Multiple Raman metrics converged on a consistent pattern, increasing confidence in the observations. The specific focus on adjacent TE adds clinically relevant detail that is often overlooked.

The compositional and structural alterations of TE have clinical implications and warrant caution for procedures involving adhesive bonding and the placement of restorative margins [22]. Recognizing a lesion-adjacent altered zone, in which enamel is visually translucent yet chemically and structurally altered, could inform conservative management and guide the development of substrate-specific adhesion protocols.

## Conclusions

MIH emerges as an alteration of enamel, in which the translucent enamel surrounding demarcated opacities is also chemically and structurally affected. Compared with healthy enamel, both adjacent translucent enamel and opaque enamel are characterized by disordered rod–interrod microarchitecture, decreased mineral crystallinity, elevated HPO_4_^2−^ and CO_3_^2−^ content, and minor organic inclusions. This combination of features is consistent with an apatite phase that is enriched in carbonate and acid phosphate and is less chemically durable in the physiological environment. The translucent enamel shows changes that are clearly distinct from healthy enamel but less pronounced than in opaque enamel, indicating that it should be regarded as affected tissue rather than normal enamel. These findings may have practical implications for bonding and margin placement in restorative dental treatments, as well as for preventive strategies.
